# Psychosocial correlates of nutritional status among people living with HIV on antiretroviral therapy: A matched case-control study in Central zone of Tigray, Northern Ethiopia

**DOI:** 10.1371/journal.pone.0174082

**Published:** 2017-03-16

**Authors:** Negassie Berhe Weldehaweria, Elsa Hagos Abreha, Meresa Gebremedhin Weldu, Kebede Haile Misgina

**Affiliations:** 1 Department of Public Health, College of Health Sciences, Aksum University, Aksum, Tigray, Ethiopia; 2 Department of Biomedical Sciences, College of Health Sciences, Semara University, Semara, Afar, Ethiopia; Istituto di Genetica Molecolare, ITALY

## Abstract

**Background:**

Malnutrition hastens progression to Acquired Human Immunodeficiency Syndromes (AIDS) related illnesses; undermines adherence and response to antiretroviral therapy (ART) in resource-poor settings. However, nutritional status of people living with HIV (PLHIV) can be affected by various psychosocial factors which have not been well explored in Ethiopia. Therefore, the objective of this study was to determine psychosocial correlates of nutritional status among people living with HIV (PLHIV) on ART in Central zone of Tigray, Northern Ethiopia.

**Methods:**

A matched case-control study design was conducted to assess psychosocial correlates of nutritional status among PLHIV on ART. Data were collected by an interviewer-administered technique using structured pre-tested questionnaire, record review using a checklist and anthropometric measurements. Cases were selected by simple random sampling and controls purposively to match the selected cases. Conditional logistic regression was used to compute relevant associations by STATA version 12.

**Results:**

The psychosocial factors independently associated with malnutrition were ever consuming alcohol after starting ART [AOR = 4.7, 95% CI: 1.8–12.3], ever smoking cigarette after starting ART [AOR = 7.6, 95% CI: 2.3–25.5], depression [AOR = 2.8, 95% CI: 1.3, 6.1], not adhering to ART [AOR = 6.8,95% CI: 2.0–23.0] and being in the second lowest wealth quintile [AOR = 4.3,95% CI: 1.1–17.7].

**Conclusion:**

Ever consuming alcohol and ever smoking cigarette after starting ART, depression, not adhering to ART and being in the second lowest wealth quintile were significantly associated with malnutrition. Therefore; policies, strategies, and programs targeting people living with HIV should consider psychosocial factors that can impact nutritional status of people living with HIV enrolled on ART.

## Introduction

Human Immunodeficiency Virus (HIV) / Acquired Human Immunodeficiency Syndromes (AIDS) is one of the most destructive epidemics the world has ever witnessed. The impact of HIV/AIDS goes beyond public health concerns. It primarily affects adult population in the productive and reproductive age groups, as such, in its endemic stage, undermines the social and economic structure of the world in general and of developing countries in particular [1]. Currently, approximately 35 million people live with HIV in the world [2,3]. Sub-Saharan Africa is the most affected region, with 25.8 million people living with HIV in 2014 [2]. Sub-Saharan Africa accounts for almost 70% of the global total of new HIV infections [2]. Ethiopia is one of the sub-Saharan countries most severely affected by the HIV/AIDS pandemic [4]. According to the 2011 demographic health survey (DHS) of Ethiopia, the overall prevalence of HIV is 1.5 nationally and 1.8% in Tigray region [5]. In 2013, there have been 793,700 people living with HIV and approximately 46,000 AIDS-related deaths in Ethiopia [4].

The rapidly expanding access to ART is changing the global HIV epidemic in momentous ways and AIDS-related mortality rates are declining rapidly. So far, the scaling up of ART averted an estimated 6.6 million AIDS-related deaths worldwide, predominantly in low- and middle-income countries [6]. In addition to prolonging life, ART increases productivity and quality of life among people living with HIV, and produce savings to the health care system through reducing the need for hospitalization [7–9]. It also has the potential to significantly reduce the risk of HIV transmission and the spread of tuberculosis [6].

However, the effectiveness of ART in achieving the intended goals can be affected by various factors. Since food is required for processing, absorption, and optimal clinical benefits for ART regimens, poor nutritional status undermines adherence and response to ART, hastens progression to AIDS-related illnesses, lowers survival time and exacerbates socioeconomic impacts [10–24]. Therefore, nutritional status is one of the factors that significantly affect the effectiveness of ART, survival status and quality of life among people living with HIV (PLHIV) [10–25].

Recent studies done in Ethiopia revealed that malnutrition is highly prevalent among people living with HIV enrolled on ART. For instance study in Hossana reported a total prevalence of 31.2%, in Dilla University referral Hospital (12.3%), in Butajira Hospital (25.2%), in Humera Hospital (42.3%), in Gondar University referral Hospital (27.8%), in Felege Hiwot Referral Hospital, Bahir Dar (25.5%), and in St. Peter Hospital, Addis Ababa (25%) [26–32].

A number of clinical and dietary factors could contribute to poor nutritional status of PLHIV as indicated by different studies conducted elsewhere [13,25–32]. On the top of clinical and dietary factors, different psychosocial factors like family support and depression and substance use which are very common among PLHIV can also affect nutritional status of PLHIV on ART directly or indirectly [26,33–38]. Therefore, information on psychosocial correlates of nutritional status among PLHIV on ART is urgently needed for prioritizing, designing and initiating intervention programs aimed at HIV care and support. However, the psychosocial correlates of nutritional status among PLHIV on ART have not been well explored in Ethiopia. Therefore, the aim of this study was to determine psychosocial correlates of nutritional status among PLHIV on antiretroviral drug treatment in Central zone of Tigray, Northern Ethiopia.

## Methods and materials

### Study design

The study has employed a matched case-control study design among people living with HIV on ART. For the comparison purpose, malnourished participants (cases) were matched by age and sex with those of well-nourished participants (controls). Age was matched within ±5 years interval. This is to mean that if a case of 28 years male was obtained, the control selected would be 28 years ±5 years old male.

### Study setting

The study was conducted in the Central zone of Tigray Region, North Ethiopia in March 2014. Tigray regional state is located in north Ethiopia with an estimated total population of 4,314,456 according to the Ethiopian Central Statistics report. According to the 2011 demographic and health survey report of Ethiopia, the estimated prevalence of HIV/AIDS in the region was about 1.8%. Among these people living with HIV, about 28,044 were on ART. Tigray regional state has 14 hospitals and 219 health centers. Central Zone, one of the seven zonal administrative divisions of Tigray regional state, had an estimated total population of 1,283,388 in 2005. Of these, 630,140 were males while the rest 653,248 were women [5]. This zone has three hospitals: St. Marry, Adwa and Abyi Adi hospitals. To get an adequate number of malnourished adults who were on ART with better documentation, two of the three hospitals (St. Marry and Adwa hospitals) were purposely selected for conducting the study considering the patient load. There were a total of 942 and 864 people living with HIV on ART in St. Marry and Adwa hospitals respectively.

### Study population

The study population was people living with HIV greater than 18 years old and who were on ART follow-up for at least one year in the selected hospitals. Cases were malnourished (BMI < 18.5kg/m^2^) people living with HIV, whereas controls were defined as people living with HIV greater than 18 years old that were on ART who were well-nourished (BMI = 18.5–25 kg/m^2)^. Participants with incomplete data for the last three visits and participants with nutritional status above optimal (BMI > 25kg/m^2^) were excluded. The sample size was computed using OpenEpi Version 3.03.17 statistical software. The assumptions for the sample size calculation were: a case: control ratio of 1:1, a two-sided significance level (alpha) of 0.05, a power of 80% and 15% difference in food insecurity between the malnourished and well-nourished subjects. The total sample size was 342 (171 cases and 171 controls). In a case-control study done in Pwani Region eastern Tanzania, the difference between the two groups in educational status, income, occupation and other psychosocial factors which also are included in our study was less than 12% [35]. However, considering the cost and the design of the study the aforementioned 15% difference assumption was taken for calculating the sample size. To select study participants, we assessed nutritional status of people living with HIV who fulfill the inclusion criteria to identify cases and controls. We have selected the cases by simple random sampling and the controls purposively to match the selected cases.

### Measurements

Data were collected by reviewing ART registration chart, interview, and anthropometric measurements. A pretested structured questionnaire adapted from different literatures and translated into the local language (Tigrigna) was used for data collection by interview. The content of the questionnaire included: factors such as age, sex, residence, marital status, economic status, occupation, smoking, alcoholism, khat (*Catha edulis)* chewing, depression, disclosing sero-status, living condition, family support, satisfaction with ART outcome, adherence, etc.

Measurement of weight was conducted using a standard beam balance that is used for weight measurement in the medical setup. The scale pointer was checked at zero before taking every measurement. Each participant was asked to remove heavy clothes. He/she stood straight and unassisted on the centre of the balance platform. Weight measurements were taken to the nearest 0.1 kg.

Height was measured using the standard scale. The subjects were asked to remove their shoes, stood erect, and positioned at the Frankfert plane with feet together and knees straight. The heels, buttocks, shoulder blades and the back of the head (occiput) were in touch against the vertical stand of the stadio meter and the values were recorded to the nearest 0.1cm.

The data were collected by two BSc nurses under the supervision of two professionals who have master degree in Health Informatics. Prior to data collection, the questionnaire was pretested on people living with HIV greater than 18 years old who were on ART in Enticho health center found in central zone of Tigray region by taking 5% (9 pairs) of the total sample size. Necessary modifications were made based on the pretest results. Training was also given to both data collectors and supervisors for three days on how to approach the study subjects collect data by the questionnaire, weight, and height measurement tools and collect data from ART registration charts. The collected data were checked out for its completeness, accuracy, and clarity by the principal investigators and supervisors. This quality checking was done daily before and after data collection and amendments were made before the next data collection process was started. Supervision was also done on the spot by principal investigators and supervisors. The overall quality checking was done at the end of the data collection process.

### Operational definitions

Nutritional status: It was estimated by computing BMI. BMI is the ratio of weight to height in meters squared. A participant was considered as well-nourished if he/she had a BMI of 18.5–25 Kg/m^2^ and as malnourished if he/she had a BMI<18.5 Kg/m^2^ [39–42].

ART adherence status: It was estimated by percent of missed dose enclosed in the last 2 month follow-up time from patient ART follow-up form combined with self-reported adherence measurement technique by asking each participant about the number of times he/she has missed taking his/her pills each month and recorded. Finally, a participant was considered as adherent if the average adherence is greater than 95% (he/she missed ≤2 doses of 30 doses or ≤3 doses of 60 doses) and non-adherent if the average adherence is less than 95% (he/she missed >2 doses of 30 doses or >3 doses of 60 doses) [43,44].

Wealth index: Economic status was assessed by using a Weighted Wealth Index incorporating household assets ownership, housing characteristics, land ownership, livestock, and electricity. Dichotomous variables were constructed and factor analysis using principal component analysis (PCA) used to reduce the 16 items. Factor loadings were used as item weights, which were totaled to yield the wealth index for each household. The total Weighted Wealth Index score was then equally divided into 5 quintiles designating fifth (highest economic status), fourth, third, second and first (lowest economic status).

Depression: Depression was assessed using a validated depression severity measure of PHQ-9 score for depression. Based on the total PHQ-9 score for depression, a score lower than 5 points was classified as not having depression and a score greater than or equal to 5 points was classified as having depression [45].

### Data processing and analysis

After coding, the data were entered using EpiInfo version 2002 and analyzed by STATA Version 12 using conditional logistic regression (Clogit) model. The association of each variable with malnutrition (BMI < 18.5kg/m^2^) was tested using odds ratio, 95% confidence interval, and p-value. A p-value of less than 0.05 was considered as statistically significant association among the different independent variables with the outcome variable.

### Ethical consideration

Ethical approval was obtained from Research and Publication Office of Aksum University and an official letter of permission was written to the respective hospitals from Tigray regional health bureau. Participants were informed about the nature of the study, its objectives, expected outcomes, and the benefits and the risks associated with it. Informed verbal consent was obtained from each study participant before data collection. Confidentiality was also ensured by using questionnaire identification numbers instead of using any personal identifiers.

## Result

### Psychosocial characteristics

A total of 340 study participants (170 pairs) were included in this study. Of the total participants included in this study, 72 (42.7%) pairs were males and 94 (57.3%) pairs were females on ART who were receiving ART care in the respective ART clinics for at least one year prior to the study. The mean age and standard deviation (SD) of the malnourished study participants were 39.3 ± 8.2 years while the mean age and standard deviation of the well-nourished study participants were 39.2 ± 7.9 years. Two-thirds of the study participants (72.9% of the well-nourished and 76.5% of the malnourished) were from urban areas. With regards to distance to ART rendering health facility from their home, 89 (52.4%) of the well-nourished and 110 (64.7%) of the malnourished participants reported that the distance to ART rendering health facility is greater than 10 kilometers from their home. More than one-third of the study participants (42.9% of the well-nourished and 34.1% of the malnourished) were married while 87 (51.2%) of the well-nourished and 85 (50%) of the malnourished participants were single. The rest 10 (5.9%) of the well-nourished and 27 (15.9%) of the malnourished participants were either divorced or widowed. Concerning to educational status, 67 (39.4%) of the well-nourished and 57 (33.5%) of the malnourished attended at least secondary school education. By occupation, nearly two-thirds were government or self-employed (53.0% of the malnourished and 72.3% of the well-nourished), one fifth were daily laborers (27.6% of the malnourished and 11.2% % of the well-nourished) while the rest had no job at all. Moreover, 61 (35.9%) of the well-nourished and 56 (32.9%) of the malnourished participants were in the 2^nd^ or in the 1^st^ lowest quintile wealth status (Table 1).

**Table 1:** Psychosocial characteristics of PLHIV on ART by their nutritional status, Central zone of Tigray, Northern Ethiopia, March 2014.

Characteristics	Malnutrition		X^2^ (P value)
	Yes	No	
Sex			1.000
Male	72 (42.4)	72(42.4)	
Female	98(57.6)	98(57.6)	
Residential address			0.454
Urban	130(76.5)	124(72.9)	
Rural	40(23.5)	46(27.1)	
Distance to hospital/clinic (km)			0.021
≤10	60(35.3)	81(47.6)	
>10	110(64.7)	89(52.4)	
Educational status			0.184
No education	45(26.5)	36(21.2)	
Elementary (1–8)	68(40)	67(39.4)	
Secondary (9–12)	44(25.9)	42(24.7)	
12+	13(7.6)	25(14.7)	
Occupation			0.000
Have no job	33 (19.4)	28 (16.5)	
Government employee	37 (21.8)	42 (24.7)	
Business/self employed	53 (31.2)	81 (47.6)	
Daily laborer	47 (27.6)	19 (11.2)	
Marital status			0.022
Single	85(50)	87(51.2)	
Married	58(34.1)	73(42.9)	
Widowed	12(7.1)	5(2.9)	
Divorced	15(8.8)	5(2.9)	
Living condition			0.013
Alone	52(30.6)	36(21.2)	
Family	86(50.6)	98(57.6)	
Parents	13(7.6)	26(15.3)	
Others	19(11.2)	10(5.9)	
Family support			0.559
Yes	140(82.4)	144(84.7)	
No	30(17.6)	26(15.3)	
Wealth index			0.002
1^st^ quintile	30(17.6)	45(26.5)	
2^nd^ quintile	26(15.3)	16(9.4)	
3^rd^ quintile	56(32.9)	40(23.5)	
4^th^ quintile	47(27.6)	40(23.5)	
5^th^ quintile	11(6.5)	29(17.1)	
Ever smoking cigarette after starting ART			0.000
Yes	66(38.8)	8(4.7)	
No	104(61.2)	162(95.3)	
Current cigarette smoking (in the last 30 days)			0.000
Yes	24(14.1)	5(2.9)	
No	146(85.9)	165(97.1)	
Ever use of alcohol after starting ART			0.000
Yes	82(48.2)	23(13.5)	
No	88(51.8)	147(86.5)	
Current alcohol consumption(in the last 30 days)			0.001
Yes	32(18.8)	11(6.5)	
No	138(81.2)	159(93.5)	
Ever khat chewing after starting ART			0.006
Yes	24(14.1)	9(5.3)	
No	146(85.9)	161(94.7)	
Current khat chewing (in the last 30 days)			0.027
Yes	16(9.4)	6(3.5)	
No	154(90.6)	164(96.5)	
Depression (based on PHQ score)			0.001
≥5 points	83(48.8)	114(67.1)	
<5 points	87(51.2)	56(32.9)	
Disclosure of their HIV sero-status			0.767
Yes	142(83.5)	144(84.7)	
No	28(16.5)	26(15.3)	
ART treatment duration (in months)			0.097
12–36	38(22.4)	51(30.0)	
37–60	84(49.4)	65(38.2)	
>60	48(28.2)	54(31.8)	
Satisfaction by ART outcome			0.244
Yes	121(71.2)	111(65.3)	
No	49(28.8)	59(34.7)	
Adherence to ART at current visit			
Adherent	130(76.5)	162(95.3)	0.000
Non-adherent	40(23.5)	8(4.7)	

Pertaining to other psychosocial aspects of the study participants, more than one-fourth (30.6% of the malnourished and 21.2% of the well-nourished) were living alone while more than half of the study participants (50.6% of the malnourished and 57.6% of the well-nourished) were living with their family. The rest study participants (26.5% of malnourished and 13.5% of the well-nourished) were living with their parents or with others. Similarly, more than three-fourths (50.6% of the malnourished and 57.6% of the well-nourished) were receiving family support. Of the total study participants included in this study; the great majority (83.5% of the malnourished and 84.7% of the well-nourished) disclosed their sero-status (Table 1).

Regarding cigarette smoking status of the study participants, 8 (4.7%) of the well-nourished and 66 (38.8%) of the malnourished participants have reported that they have history of cigarette smoking, after starting ART. The result also showed that 5 (2.9%) of the well-nourished and 24 (14.1%) of the malnourished participants have reported that they are smoking currently. Similarly, 23 (13.5%) of the well-nourished and 82 (48.2%) of the malnourished participants have reported that they have history of alcohol consumption after starting ART. It is also shown that 32 (18.8%) of the malnourished and 11 (6.5%) of the well-nourished are consuming alcohol currently. Furthermore, 24 (14.1%) of the malnourished and 9 (5.3%) of the well-nourished participants have reported that they have history of khat chewing after starting ART. It is also shown that 16 (9.4%) of the malnourished and 6 (3.5%) of the well-nourished participants are chewing khat currently. Nearly one-third of the well-nourished 56 (32.9%) and more than half of the malnourished 87 (51.2%) participants were depressed (Table 1).

Of the total study participants included in this study, more than two-thirds (65.3% of the malnourished and 65.3% of the well-nourished) were satisfied with ART outcome. Concerning to the adherence to ART, the results of the current study showed that 40 (23.5%) of the well-nourished and 8 (4.7%) of the malnourished participants were not adherents while the rest were adherents (Table 1).

### Psychosocial correlates of nutritional status

In the multivariate analysis used to determine psychosocial correlates of nutritional status among PLHIV on ART, ever smoking cigarette after starting ART, ever use of alcohol after starting ART, depression, second lowest wealth quintile compared with the fifth quintile (highest) and not adhering to ART were significantly associated with malnutrition. People living with HIV on ART who ever used alcohol after starting ART were 4.7 times more likely malnourished than their matched counterparts [AOR = 4.7, 95% CI: 1.8, 12.3]. Likewise, people living with HIV on ART who ever smoked cigarette after starting ART were 7.6 times more likely malnourished than their matched counterparts [AOR = 7.6, 95% CI: 2.3–25.5]. People living with HIV on ART who were depressed were 2.8 times more likely malnourished than their matched counterparts [AOR = 2.8, 95% CI: 1.3, 6.1]. In addition, people living with HIV who were non-adherent to ART [AOR = 6.8, 95% CI: 2.0–23.0] and those who were in the second lowest wealth quintile [AOR = 4.3, 95% CI: 1.1–17.7] were 6.8 and 4.3 times more likely malnourished than their matched counterparts (Table 2).

**Table 2:** Psychosocial correlates of nutritional status among adult PLHIV on ART in Central zone of Tigray, Northern Ethiopia, March 2014.

Characteristic	Malnutrition		COR (95% CI)	AOR (95% CI)
	Yes	No		
Educational status				
No education	45(26.5)	36(21.2)	2.8 (1.2–6.9)	1.2 (0.3–4.6)
Elementary (1–8)	68(40)	67(39.4)	2.2 (1.0–5.1)	1 (0.3–3.4)
Secondary (9–12)	44(25.9)	42(24.7)	2.2 (1.0–5.2)	0.9 (0.2–3.5)
12+	13(7.6)	25(14.7)	1	1
Occupation				
Have no job	33 (19.4)	28 (16.5)	1	
Government employee	37 (21.8)	42 (24.7)	0.8 (0.4–1.5)	
Business/self employed	53 (31.2)	81 (47.6)	0.6 (0.3–1.1)	
Daily laborer	47 (27.6)	19 (11.2)	2.1 (1.0–4.6)	
Marital status				
Single	85(50)	87(51.2)	1	
Married	58(34.1)	73(42.9)	0.9 (0.5–1.4)	
Widowed	12(7.1)	5(2.9)	4.3 (1.03–17.9)	
Divorced	15(8.8)	5(2.9)	5.2 (1.4–20.3)	
Living condition				
Alone	52(30.6)	36(21.2)	1	1
Family	86(50.6)	98(57.6)	0.6 (0.3–1.01)	0.6 (0.3–1.5)
Parents	13(7.6)	26(15.3)	0.3 (0.2–0.8)	0.4 (0.1–1.7)
Others	19(11.2)	10(5.9)	1.4 (0.5–3.8)	1.8 (0.3–10.8)
Wealth index				
1^st^ quintile(lowest)	30(17.6)	45(26.5)	1.7 (0.7–4.1)	1.4 (0.4–5.3)
2^nd^ quintile	26(15.3)	16(9.4)	3.6 (1.4–9.2)	4.3 (1.1–17.7)
3^rd^ quintile	56(32.9)	40(23.5)	3.4 (1.5–7.8)	3 (1.0–9.7)
4^th^ quintile	47(27.6)	40(23.5)	2.9 (1.3–6.9)	2.5 (0.7–9.3
5^th^ quintile(highest)	11(6.5)	29(17.1)	1	1
Ever cigarette smoking after starting ART				
Yes	66(38.8)	8(4.7)	10.7 (4.6–24.6)	7.6 (2.3–25.5)
No	104(61.2)	162(95.3)	1	1
Ever use of alcohol after starting ART				
Yes	82(48.2)	23(13.5)	6.9 (3.6–13.4)	4.7 (1.8–12.3)
No	88(51.8)	147(86.5)	1	1
Ever khat chewing after starting ART				
Yes	24(14.1)	9(5.3)	2.7 (1.2–5.7)	0.4 (0.1–1.9)
No	146(85.9)	161(94.7)	1	1
Depression (based on PHQ-9 score)				
≥5 points	83(48.8)	114(67.1)	1	1
<5 points	87(51.2)	56(32.9)	2.4 (1.5–4.0)	2.8 (1.3–6.1)
Distance to hospital/clinic (kms)				
≤10	60(35.3)	81(47.6)	1	1
>10	110(64.7)	89(52.4)	1.7 (1.1–2.7)	2.1 (0.9–4.6)
ART treatment duration (in months)				
12–36	38(22.4)	51(30.0)	1	1
37–60	84(49.4)	65(38.2)	1.9 (1.1–3.3)	1.3 (0.5–1.5)
>60	48(28.2)	54(31.8)	1.1 (0.7–2.0)	0.8 (0.3–1.9)
Adherence to ART at current visit				
Adherent	130(76.5)	162(95.3)	1	1
Non-adherent	40(23.5)	8(4.7)	5.6 (2.5–12.5)	6.8 (2.0–23.0)

## Discussion

This study focused on determining psychosocial correlates of nutritional status of adult people living with HIV taking antiretroviral therapy in health facilities of central zone of Tigray Regional State. A total of 340 (170 malnourished and 170 well-nourished) participants were included in this matched case-control study. Cases (malnourished participants) and controls (well-nourished participants) were matched by age and sex. Of the total matched study participants, 72 (42.7%) pairs were males and 98 (57.3%) pairs were females who were on ART for at least one year prior to the study.

Results from this study showed that ever consumption of alcohol and ever smoking cigarette after starting ART, depression, not adhering to ART and second lowest wealth quintile were significantly associated with malnutrition among people living with HIV enrolled in ART.

In this study alcohol consumption was significantly associated with malnutrition among people living with HIV enrolled on ART. The risk of malnutrition among people living with HIV enrolled in ART who ever used alcohol was almost five times higher when compared to their counterparts. This lends support to a more recent study done by Molina et al [46], indicating that chronic alcohol abuse is associated with immunosuppressive effects that exacerbate the HIV-related immune-suppression. This accelerates disease progression which can eventually result in malnutrition. This is also supported by Gregory et al and other similar studies [46–50]. This could be due to the well-established fact that alcohol use alters the metabolism of vitamins and minerals leading to poor nutritional outcomes [46,48,50]. On top of that, it may be due to the negative effect of alcohol on dietary intake due to reduced appetite [46,47]. Similarly, people living with HIV who drink alcohol have an increased risk of gastrointestinal tract problems like aphthous ulcer and candidiasis that could negatively affect their nutritional status [46–48,50]. On the other way round, it could also be due to the negative effect of alcohol consumption on ART adherence [20,33,46,47,49,51–54], which can ultimately result in bad diseases prognosis and malnutrition [50,54]. The implication of this finding is mainly to implement a strategic intervention toward HIV-infected patients who have an alcohol use background in order to provide them appropriate health care, psychological support and improve ART outcomes.

The odds of malnutrition after starting ART among people living with HIV who ever smoked cigarette was nearly eight-fold higher when compared to their matched counterparts. This is in line with the finding of a study conducted by Petrosillo and Cicalini in Rome [55], indicating that smoking confers different mortality risks in HIV-positive patients which can result in significantly increased mortality among HIV-positive patients who smoke than their counterparts as evidenced by several studies [56–60]. Malnutrition may be one of the risks that cause greater morbidity and mortality risk among HIV-positive patients who smoke. Malnutrition among HIV-positive patients who smoke may be related to the fact that smokers encounter reduced appetite and disrupted body’s natural energy balance [46,47]. Similarly, HIV-positive patients who smoke have an increased risk of different gastrointestinal tract problems that can lower dietary intake and eventually result in malnutrition [56,61]. This is also supported by the finding from a study among HIV-infected Japanese men which identified reducing smoking as an imperative strategy in enhancing disease management efforts by reducing co-morbidities like malnutrition that increase mortality risk [60]. It could be also explained due to the association of smoking and other substance use such as alcohol, khat, cocaine, heroin marijuana etc [36–38,47], which can have synergetic effect on nutritional status of people living with HIV on ART. On the other way round, smoking in HIV-infected patients increase risk of getting bacterial pneumonia, pulmonary tuberculosis, pneumocystis carinii pneumonia (PCP), oral and esophageal candidiasis and cardiovascular disorders [56,58,61–63], which in turn may result in malnutrition. This suggests that HIV care and support programs should address psychosocial factors.

Depression was significantly associated with malnutrition among people living with HIV enrolled on ART in this study. Among those people living with HIV enrolled on ART who were depressed, the risk of malnutrition was almost three-folds higher compared to their matched counterparts. According to Leserman J et al depression accelerates disease progression among people living with HIV [64], which can affect their appetite, food security status and eventually results in malnutrition in particular and lower quality of life in general [65–69]. It may be also due to the association of depression and substance use such as alcohol, khat, cigarette smoking, etc [36–38], which can have synergetic effect of on nutritional status of people living with HIV on ART. On the other way round, malnutrition may also result in depression which in turn can exacerbate the nutritional status. This finding suggests a need to establish and strengthen programs that target psychosocial problems and nutritional status among people living with HIV enrolled on ART.

Furthermore, people living with HIV enrolled on ART who were non-adherent to ART were about 6.8 times at risk of malnutrition compared to their matched counterparts. This may be due to the negative impact of poor ART adherence on viral suppression and disease progression which may eventually result in malnutrition [10,20,47]. On the other way round, results from other similar studies also indicated that malnutrition is associated with non-adherence to ART among HIV-infected individuals on ART [18]. Another similarly matched case-control study by Berhe et al in Ethiopia, showed that people living with HIV enrolled on ART who were malnourished were far more likely to be non-adherent to ART compared to their matched counterparts [13]. This is strengthened further by findings a of study from Zambia which indicated that clinic-based food assistance is associated with increased medication adherence among HIV-infected adults on long-term antiretroviral therapy [19,20]. So, both malnutrition and poor adherence can affect one another and their synergetic effect may result in poor viral suppression [10,20]. The implication is adherence to ART and nutritional status of people living with HIV enrolled on ART should be considered in the HIV care and support programs.

Those people living with HIV enrolled on ART in the 2^nd^ quintile wealth status were 4.3 times more likely malnourished than those in the 5^th^ quintile wealth status. This is supported by the study from Democratic Republic of Congo which indicated poor social class was associated with malnutrition among people living with HIV enrolled on antiretroviral treatment [70]. Evidence obtained from a clinical cohort in Uganda also indicated that socioeconomic factors are determinants of poverty and then mortality among people living with HIV enrolled on ART [71]. Similarly, very poor economic status was significantly associated with malnutrition in a study done in Dilla University Referral Hospital and in Gondar Referral Hospital, Ethiopia [27,30]. This may be due to the fact that poor socioeconomic status leads to lack of access to food resulting in malnutrition.

Generally, in addition to the negative consequences of clinical and dietary factors on nutritional status of people living with HIV enrolled on ART shown in other studies, this study elucidated psychosocial correlates of nutritional status among people living with HIV enrolled on ART care. However, this study has its own drawbacks. Our study design which is observational design provides statistical associations of psychosocial factors with nutritional status which cannot establish whether low BMI followed or preceded psychosocial factors studied. Since the study addressed some socially sensitive questions, in order to obtain a reliable data, respondents were well informed about the purpose of the study and they were reassured about confidentiality and anonymity. However, this study is still not free from perceived social desirability of responses and recall biases. Moreover, even if efforts were made to address important psychosocial aspects, it was not possible to gather comprehensive data which include all relevant factors exhaustively like the amount of alcohol and number of cigarettes taken in a specified period.

## Conclusion

This study determined ever consuming alcohol and ever smoking cigarette after starting ART, depression, not adhering to ART and being in the second lowest wealth quintile as significantly associated with malnutrition among people living with HIV enrolled on ART. Therefore Policies, strategies, and programs targeting people living with HIV should consider psychosocial factors that can impact nutritional status of people living with HIV enrolled on ART. Short and long term nutritional interventions targeting people living with HIV enrolled on ART also should focus on those with psychosocial problems especially in food and nutrition insecure areas. Another study that can address all relevant dimensions of the psychosocial issue is recommended.
